# Role of CD45 Signaling Pathway in Galactoxylomannan-Induced T Cell Damage

**DOI:** 10.1371/journal.pone.0012720

**Published:** 2010-09-14

**Authors:** Eva Pericolini, Elena Gabrielli, Giovanni Bistoni, Elio Cenci, Stefano Perito, Siu-Kei Chow, Francesca Riuzzi, Rosario Donato, Arturo Casadevall, Anna Vecchiarelli

**Affiliations:** 1 Microbiology and Anatomy Sections, Department of Experimental Medicine and Biochemical Sciences, University of Perugia, Perugia, Italy; 2 Department of Plastic and Reconstructive Surgery, University of Rome “La Sapienza” Medical School, Rome, Italy; 3 Department of Microbiology and Immunology of the Albert Einstein College of Medicine, Bronx, New York, United States of America; University of Toronto, Canada

## Abstract

Previously, we reported that Galactoxylomannan (GalXM) activates the extrinsic and intrinsic apoptotic pathways through an interaction with the glycoreceptors on T cells. In this study we establish the role of the glycoreceptor CD45 in GalXM-induced T cell apoptosis, using CD45^+/+^ and CD45^−/−^ cell lines, derived from BW5147 murine T cell lymphoma. Our results show that whereas CD45 expression is not required for GalXM association by the cells, it is essential for apoptosis induction. In CD45^+/+^ cells, CD45 triggering by GalXM reduces the activation of Lck, ZAP70 and Erk1/2. Conversely, in CD45^−/−^ cells, Lck was hyperphosphorylated and did not show any modulation after GalXM stimulation. On the whole, our findings provide evidence that the negative regulation of Lck activation occurs via CD45 engagement. This appears to be related to the capacity of GalXM to antagonize T cell activation and induce T cell death. Overall this mechanism may be responsible for the immune paralysis that follows GalXM administration and could explain the powerful immunosuppression that accompanies cryptococcosis.

## Introduction

CD45 is a type 1 transmembrane molecule found on the surface of all nucleated hematopoietic cells and their precursors, except for mature erythrocytes and platelets. It is one of the most abundant cell surface glycoproteins, comprising up to 10% of the cell surface area. The cytoplasmic region shares a remarkable 95% homology across all mammalian species analyzed. In contrast, the extracellular domain manifests only 35% homology [1], [2]. CD45 is expressed in various isoforms ranging in molecular weight from 180–235 kDa that arise from cell type-specific alternative splicing of variable exons (exons 4/A, 5/B, 6/C and 7) encoding sequences at the NH_2_-terminal domain [3], [4]. Expression of different CD45 isoforms is cell type specific, and depends on the state of activation and differentiation of hematopoietic cells [3]. B lymphocytes express the high molecular weight isoform of 220 kDa (also termed B220), which includes all alternatively spliced CD45-exons (CD45RABC). Immature CD4^+^ CD8^+^ thymocytes express mainly low molecular weight CD45 isoforms, whereas mature CD4^+^ or CD8^+^ thymocytes and peripheral CD4^+^ CD8^+^ T cells can express multiple isoforms [5]. Expression of different CD45 isoforms also changes during T cell activation and “naïve” T cells switch from high molecular weight (CD45RB) to low molecular weight CD45 (CD45RO) isoforms upon stimulation. CD45 isoform patterns can also change in response to cytokine [6]. Differential expression of CD45 splice variants has frequently been used to distinguish between “naïve” CD45RB and “memory” CD45RO T cells. However, this phenotypic distinction parallels the state of activation of a given T cell: the CD45RO memory phenotype is reversible because CD45RO cells can re-express high molecular weight CD45 isoforms [7]. Moreover, the expression patterns for a given population are not absolute and a single cell type can express multiple CD45 isoforms [2]. CD45 has an intrinsic tyrosine phosphatase activity and has been implicated in cell proliferation, signaling and differentiation and is associated with the B cell receptor during signaling [8].

Galactoxylomannan (GalXM), is a minor component of capsular material of *Cryptococcus neoformans* (*C. neoformans*) which is an ubiquitous encapsulated yeast that causes disease predominantly in immunocompromised hosts [9]. GalXM has an α-(1–6) galactan backbone containing four potential short oligosaccharide branch structures. The branches are 3-O-linked to the backbone and consist of an α-Man-(1–3)-α-Man-(1–4)-β-Gal trisaccharide with variable amounts of β-(1–2) or β-(1–3) xylose side groups [10], [11]. A recent study has also confirmed the presence of glucuronic acid in the side chain [12]. GalXM has been shown to induce TNF-α production from peripheral blood mononuclear cell (PBMC) [13] and different cytokine profiles in RAW 264.7 macrophages [14].

De Jesus *et al*. recently investigated the immunological and biological effects of GalXM in mice and reported that GalXM immunization elicits a state of immunological paralysis in mice characterized by the disappearance of Ab-producing cells in spleen [15]. In a previous paper we demonstrated that GalXM affected selected immune responses, including a significant impairment of T cell proliferation, an increase in IFN-γ and IL-10 production, Fas and FasL upregulation and induction of apoptosis of T lymphocytes [16]. Moreover, in a recent study we demonstrated that GalXM promoted apoptosis of T and Jurkat cells by interacting with the glycoreceptors such as CD7, CD43 and CD45. In particular we showed that GalXM-induced apoptosis was primarily mediated by CD45 crosslinking. This observation implies that a microbial compound can directly affect T cell function by bypassing processing by antigen presenting cells (APC) [17].

To investigate the requirement of CD45 expression in T cell responses influenced by GalXM, we used CD45^+/+^ and CD45^−/−^ cell lines derived from BW5147 murine T cell lymphoma [18] and found that CD45 expression was required for the modulation of T cell death and proliferation induced by GalXM.

## Materials and Methods

### Reagents and Media

Dulbecco's Modified Eagle's Medium (DMEM) with 4 mM L-glutamine was obtained from American Type Culture Collection (ATCC) (Manassas, VA). Fetal calf serum (FCS) was purchased from Gibco BRL (Paisley, Scotland). Penicillin-streptomycin solution was obtained from Sigma-Aldrich (St. Louis, MO). R-phycoerythrin (RPE)-conjugated mouse monoclonal antibody (mAb) to CD45 (Rat IgG1k isotype) was obtained from ImmunoTools GmbH (Friesoythe, Germany). 5-DTAF [5-(4,6-Dicholotriazinyl) aminofluorescein] (5-DTAF) was purchased from Chem Progress s.r.l. (Sesto Ulteriano, MI, Italy). Purified mAb to CD3 (Hamster IgG1k isotype) was obtained from ImmunoTools. Rabbit polyclonal antibodies to phospho-Lck (Tyr505) and Lck were obtained from Cell Signaling Technology (Beverly, MA). Rabbit polyclonal antibodies to phospho-Erk1/2 (Thr202), Erk1/2 (C-16), phospho-ZAP70 (Tyr 493) and actin (H-300) were obtained from Santa Cruz Biotechnology (Delaware Avenue, CA). CD45 tyrosine phosphatase inhibitor BN82002 hydrochloride salt (BN82002) was obtained from Sigma-Aldrich. The Csk inhibitor (ASN 05260638) was obtained from Asinex (Moscow, Russia). Cy3 labelled conjugated affinity purified secondary antibody was obtained from Chemicon Int. (Temecula, CA). Phytohemagglutinin (PHA) and isotype controls were obtained from Sigma-Aldrich. All reagent and medium were negative for endotoxin, as assessed by *Limulus* amebocyte lysate assay (QCL-1000, BioWhittaker).

### Cryptococcal polysaccharide

GalXM from *C. neoformans* was purified as described elsewhere [19]. To eliminate LPS contamination in the GalXM preparation, lyophilized GalXM was reconstituted in 1 X phosphate buffer saline (PBS) and dialyzed in Endosafe LPS-free water for 3 weeks until the dialysate resulted negative using *Limulus* amebocyte assay, as previously described [15]. GalXM was checked again for LPS contamination after reconstitution and resulted negative at the threshold of the assay (<0.50 EU/ml).

### Preparation of fluorescein-labelled GalXM (GalXM-FLUOS)

A fluorescein derivative of GalXM (GalXM-FLUOS) was prepared by incubating GalXM with 5-DTAF according to study done by De Belder A.N. *et al.* [20]. The GalXM-FLUOS was separated from free 5-DTAF by ethanol precipitation and was solubilised in PBS.

### Cell lines

Murine T cell lymphoma cell line BW5147 (CD45^+/+^) and the mutant cell line BW5147 (T200^−^) (CD45^−/−^), derived from BW5147 cells, were purchased from ATCC. Both types of BW5147 cells used in this study are TCR positive [21]. More specifically, these cells express some CD3 subunits, such as ε, γ and small amounts of δ, but lack a CD3 ζ chain, thus preventing their TCR α and β chains from reaching the cell surface. Nevertheless, upon CD3 stimulation these cell lines readily respond to mAb to CD3 triggering, since there is a small amount of CD3 ε-γ heterodimers that reach the surface and signal via their ITAMs [22], [23]. Cells were maintained in DMEM supplemented with 10% FCS (complete medium) and antibiotics (100 U/ml penicillin and 100 µg/ml streptomycin) at 37°C and 5% CO_2_.

### Flow cytometry analysis

BW5147 and BW5147 (T200^−^) cells (both 1×10^6^/ml) were incubated for 30 min or 2 h in the presence or absence of GalXM-FLUOS (10 µg/ml) in complete medium at 37°C and 5% CO_2_. In selected experiments, cells (both 1×10^6^/ml) were pre-treated for 5 min in the presence or absence of GalXM (10 µg/ml) and subsequently incubated for 5 min in the presence or absence of GalXM-FLUOS (10 µg/ml) as previously described. After incubation, cells were washed twice, fixed with 4% formalin for 10 min at room temperature (RT), washed and resuspended in 0.5 ml of fluorescence buffer (FB) and analyzed by flow cytometry using FACScan flow cytofluorometer (BD Biosciences). Results shown are from one representative experiment of five independent experiments with similar results. BW5147 and BW5147 (T200^−^) cells (both 1×10^6^/ml) were incubated for 30 min, 2 or 18 h in the presence or absence of GalXM (10 µg/ml) in complete medium at 37°C and 5% CO_2_. In selected experiments, cells (both 1×10^6^/ml) were incubated for 30 min in the presence or absence of GalXM-FLUOS (10 µg/ml) as previously described. After incubation, cells were washed, fixed with 4% formalin for 10 min at RT, washed and reacted with RPE-labelled mAb to CD45 (2 µl/tube) for 40 min on ice. After incubation, cells were washed twice with FB, resuspended in 0.5 ml of FB and analyzed by flow cytometry. Data are expressed as mean of fluorescence intensity (MFI) of labelled cells and shown as FACScan histrograms or analyzed by two-color flow cytometry and the results shown are from one representative experiment of five independent experiments with similar results. To evaluate phospho-ZAP70 activation, cells (both 1×10^6^/ml) were pre-activated in the presence or absence of PHA (10 µg/ml) for 30 min in complete medium at 37°C and 5% CO_2_ and then incubated for 30 min in the presence or absence of GalXM (10 µg/ml) or BN82002 (6 µM) [24], [25] as previously described. After incubation, cells were washed, fixed with 1.5% formalin for 10 min at RT, washed, incubated with absolute methanol (500 µl/10^6^ cells) for 10 min on ice to permeable cells, washed twice with FB and incubated with rabbit polyclonal Ab to phospho-ZAP70 (dilution 1∶50, Santa Cruz Biotechnology Inc.) followed by Cy3 labelled conjugated affinity purified secondary antibody (dilution 1∶100, Chemicon Int.) [26]. Data are expressed as MFI of labelled cells. Autofluorescence was assessed using untreated cells. Control staining of cells with irrelevant antibody was used to obtain background fluorescence values.

### Fluorescence microscopy

BW5147 and BW5147 (T200^−^) cells (both 1×10^6^/ml) were incubated for 30 min in the presence or absence of GalXM-FLUOS (10 µg/ml) in complete medium at 37°C and 5% CO_2_. After incubation, cells were washed and counterstained with Evans' Blue (StemCell Technologies Inc., Milan, Italy), and subsequently examined under fluorescent light microscopy (Carl Zeiss, Jena, Germany). To study the association of GalXM to CD45, cells (1×10^6^/ml) were incubated for 30 min or 2 h in the presence of GalXM-FLUOS or, in selected experiments, in the presence or absence of GalXM (10 µg/ml) in complete medium at 37°C and 5% CO_2_. After incubation, cells were washed, fixed with absolute methanol (1 ml/tube) for 10 min at RT and then labelled with RPE mAb to CD45 for 40 min at 4°C. After staining, cells were washed twice with FB, resuspended in FB, collected by cytospin (2×10^5^/200 µl) at 700 g for 7 min, reacted with 4′6-diamidino-2-phenylindole (DAPI, Sigma) and subsequently examined under fluorescent light microscopy (Carl Zeiss). Irrelevant antibodies were used to obtain background of fluorescence. Each condition was studied in triplicate, and three images were taken for each sample. The figures shown are representative.

### Evaluation of apoptosis

BW5147 and BW5147 (T200^−^) cells (both 1×10^6^/ml) were incubated for 18 h in the presence or absence of PHA (10 µg/ml), mAb to CD3 (1 µg/ml) or GalXM (10 µg/ml) in complete medium at 37°C and 5% CO_2_. In selected experiments, cells (both 1×10^6^/ml) were cultured in the presence or absence of Csk inhibitor (ASN 05260638) [27] in complete medium for 10 min at 37°C plus 5% CO_2_. After incubation, cells were incubated for 18 h in the presence or absence of GalXM (10 µg/ml) in complete medium at 37°C and 5% CO_2._ After stimulation, cells were centrifuged, resuspended in hypotonic propidium iodide (PI) solution (50 µg/ml) (Sigma-Aldrich), and kept for 1 h at RT. The percentage of cells undergoing apoptosis was evaluated by flow cytometry analysis as previously described [28]. Data are expressed as fold increase in the percentage of apoptotic cells and/or shown as FACScan histograms. In selected experiments, BW5147 and BW5147 (T200^−^) cells (both 1×10^6^/ml) were incubated as described above. After incubation, cells (2×10^5^/200 µl) were collected by cytospin (700 g for 7 min) and stained by Hemacolor.

### Western blot analysis

BW5147 and BW5147 (T200^−^) cells (both 5×10^6^/ml) were pre-treated for 30 min in the presence or absence of mAb to CD3 (1 µg/ml) in complete medium at 37°C and 5% CO_2._ After incubation, cells were washed with DMEM, resuspended in complete medium and stimulated for 10 or 30 min in the presence or absence of GalXM (10 µg/ml) or BN82002 (6 µM) at 37°C and 5% CO_2._ After incubation, cells were washed and lysed with mammalian protein extraction reagent in the presence of protease and phophatase inhibitors (all from Pierce, Rockford, IL). Protein concentration were determined with a BCA protein Assay Reagent kit (Pierce). The lysates (30 µg of each sample) were separated by sodium dodecyl-sulfate-10% PAGE, transferred to a nitrocellulose membrane (Pierce) for 1 h at 100 V in a blotting system (Bio-Rad, Hercules, CA) for Western blot analysis, and the membranes were incubated overnight with rabbit polyclonal Abs to phospho-Lck (dilution 1/1000; Cell Signaling Technology) and phospho-Erk1/2 (dilution 1/200; Santa Cruz Biotechnology Inc) in blocking buffer. Detection was achieved with the appropriate secondary Abs coupled to HRP, followed by Chemilucent Trial Kit (Chemicon Int., Temecula, CA). Immunoblotting with rabbit polyclonal Abs to Lck (dilution 1/1000; Cell Signaling Technology) and Erk1/2 (dilution 1/200; Santa Cruz Biotechnology Inc.) were used as internal loading controls to ensure equivalent amounts of protein in each lane. Immunoreactive bands were visualized and quantified by Chemidoc Instrument (Bio-Rad).

### Evaluation of proliferation

BW5147 and BW5147 (T200^−^) cells (both 1×10^6^/ml) were pre-treated for 30 min in the presence or absence of PHA (10 µg/ml) in complete medium at 37°C and 5% CO_2._ After incubation, cells were washed with DMEM, resuspended in complete medium and stimulated for 48 h in the presence or absence of GalXM (10 µg/ml) or BN82002 (6 µM) at 37°C and 5% CO_2._ After incubation, cell proliferation was evaluated by the use of an ATP bioluminescence kit (Via Light kit, Lonza Rockland Inc., ME). Briefly, 100 µl of each sample was added to a 96-well culture plate, 50 µl of lysis reagent was added to each well, and after 10 min of incubation, 100 µl of ATP monitoring reagent (AMR Plus) were added to each sample. After 2 min of incubation at RT, luminescence was measured by a luminometer (Infinite 200, Tecan).

### Statistical analysis

Data are reported as the mean ± s.e.m. from duplicate samples of 5–7 experiments and were evaluated by ANOVA. Post hoc comparisons were done with Bonferroni's test. A value of *p*<0.05 was considered significant.

## Results

**Figure 1:**
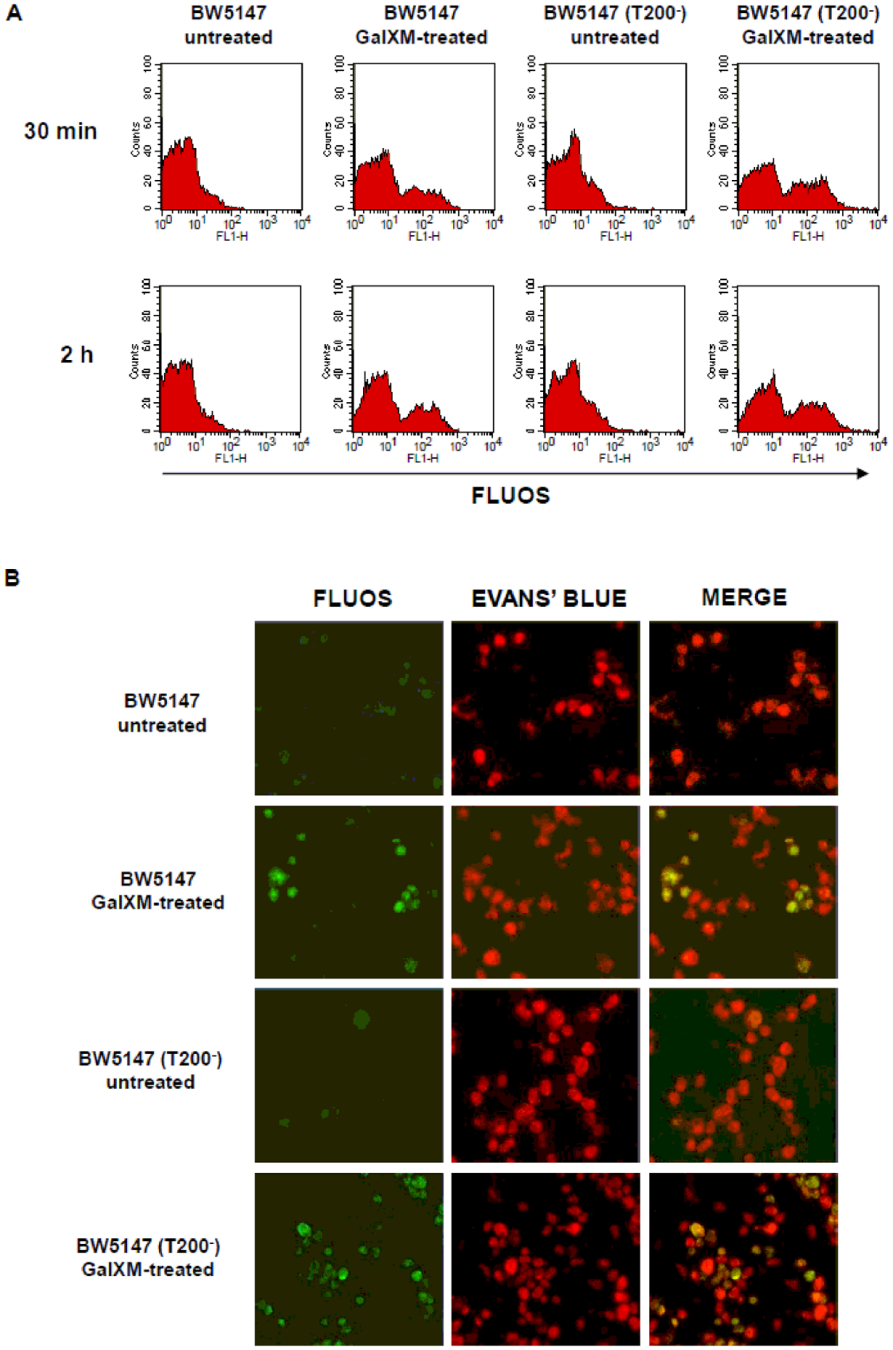
GalXM association by BW5147 cells. (**A**) BW5147 and BW5147 (T200^−^) cells (both 1×10^6^/ml) were incubated for 30 min or 2 h in the presence or absence of GalXM-FLUOS (10 µg/ml). After incubation, cells were analyzed by FACScan flow cytometry. Fluorescence histograms of positive cells, from one representative experiment out of five with similar results, were reported. (**B**) In selected experiments, cells were incubated for 30 min as described above and subsequently examined under fluorescent light microscopy. Evans' Blue was used as a counterstain. Note the green fluorescence of GalXM-treated cells.

**Figure 2:**
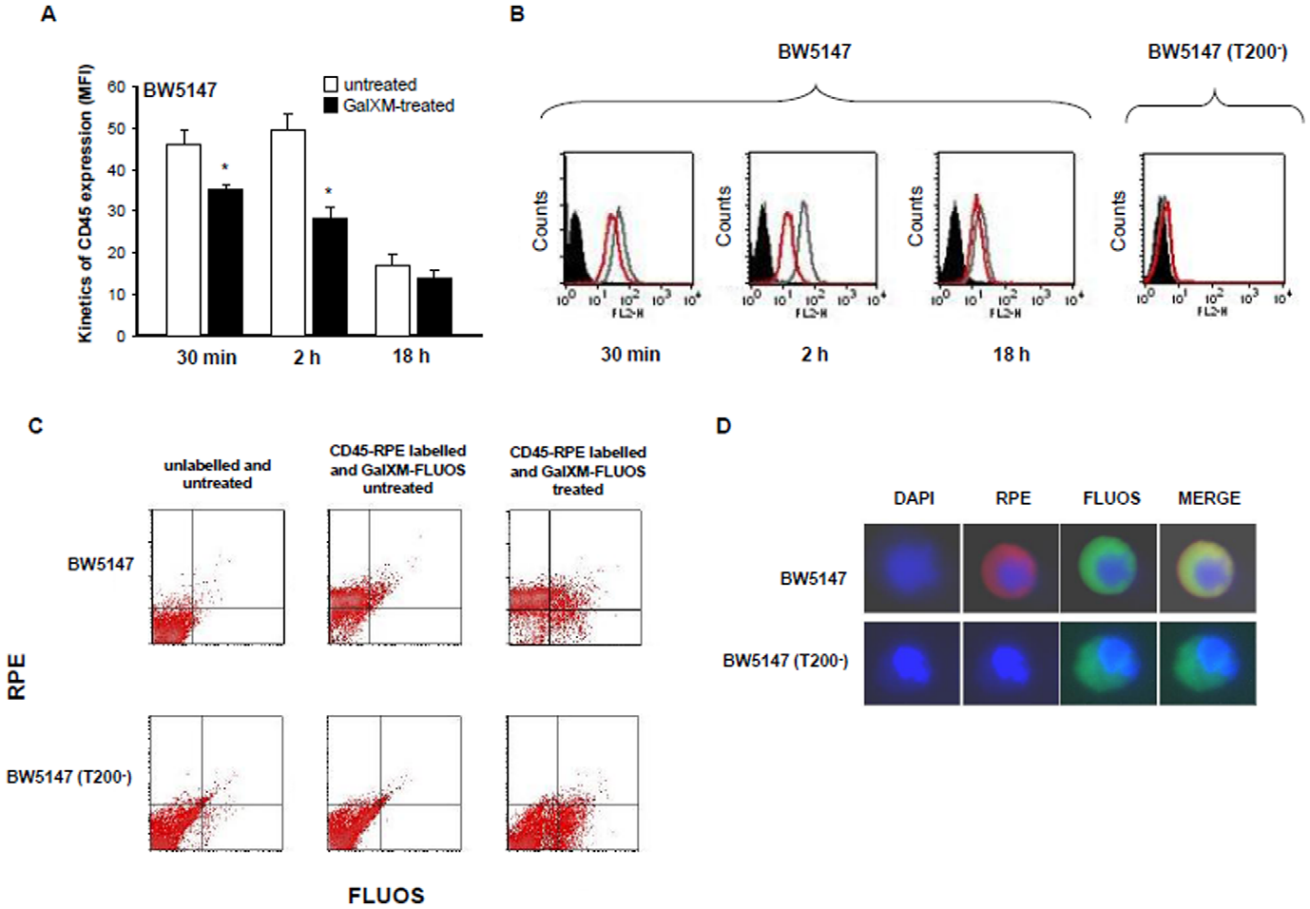
CD45 expression on BW5147 cells treated with GalXM and GalXM colocalization with CD45. BW5147 and BW5147 (T200^−^) cells (both 1×10^6^/ml) were incubated for 30 min, 2 h or 18 h in the presence or absence of GalXM (10 µg/ml). After incubation, cells were labelled with RPE mAb to CD45 and analyzed by FACScan flow cytometry. The mean of fluorescence intensity (MFI) of labelled cells is shown as a histogram (**A**) or is shown as FACScan histograms (**B**). Error bars denote s.e.m. *, *p*<0.05 (GalXM-treated *vs* untreated, n = 7). In selected experiments, cells (1×10^6^/ml) were incubated for 30 min in the presence or absence of GalXM-FLUOS (10 µg/ml). After incubation, cells were labelled with RPE mAb to CD45 and analyzed by FACScan flow cytometry. Dot plots of the percentage of double positive cells, from one representative experiment out of five with similar results, were reported (**C**). Cells (1×10^6^/ml) were incubated for 30 min with GalXM-FLUOS (10 µg/ml) (green), labelled with RPE mAb to CD45 (red), stained with DAPI (blue) and subsequently examined under fluorescent light microscopy (D).

**Figure 3:**
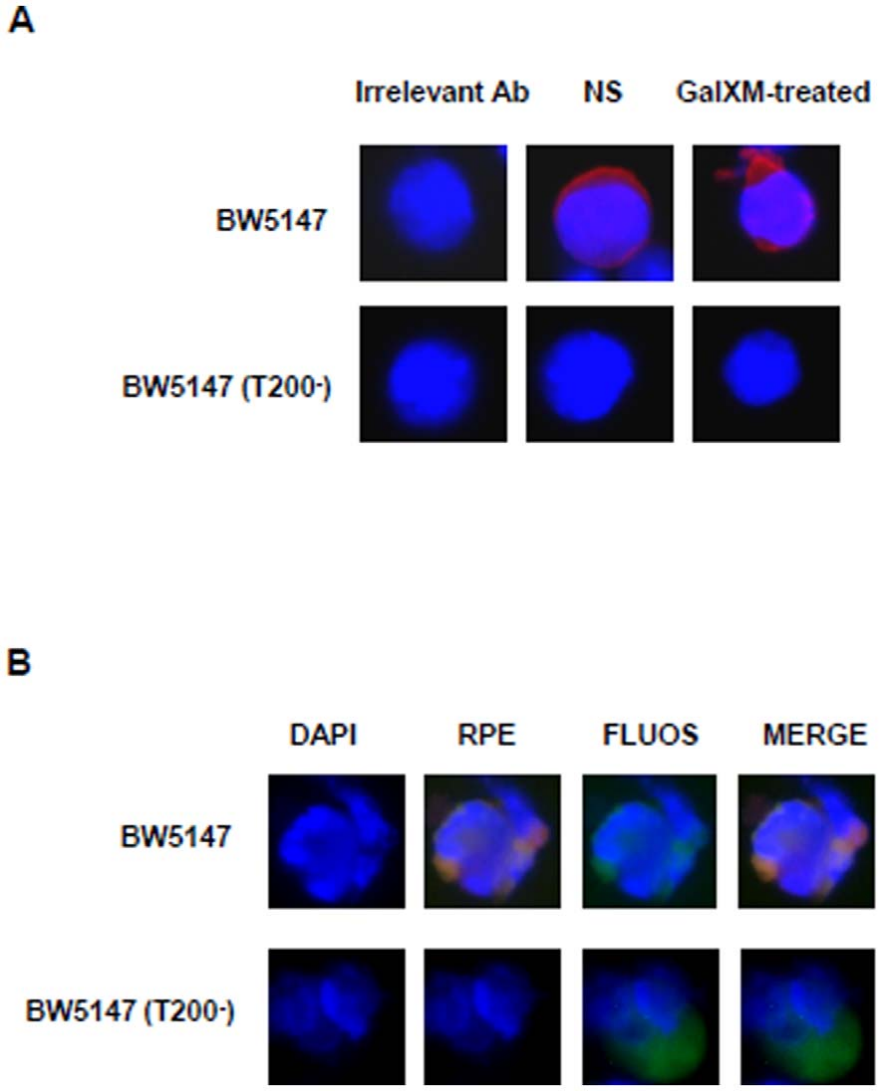
Segregation of CD45 GalXM-induced on BW5147 cells. Fluorescence microscopy analysis of BW5147 and BW5147 (T200^−^) cells incubated for 2 h in the presence or absence (NS) of GalXM (**A**) or GalXM-FLUOS (green) (**B**) (both 10 µg/ml). After incubation, cells were labelled with RPE mAb to CD45 (red) and then examined under fluorescent light microscopy in the presence of DAPI (blue). Note the CD45 segregation in BW5147 cells treated with GalXM (**A**). The colocalization of GalXM with CD45 and the receptor segregation on BW5147 cells was demonstrated in the merged image of panel B (**B**). Original magnification 100x.

**Figure 4:**
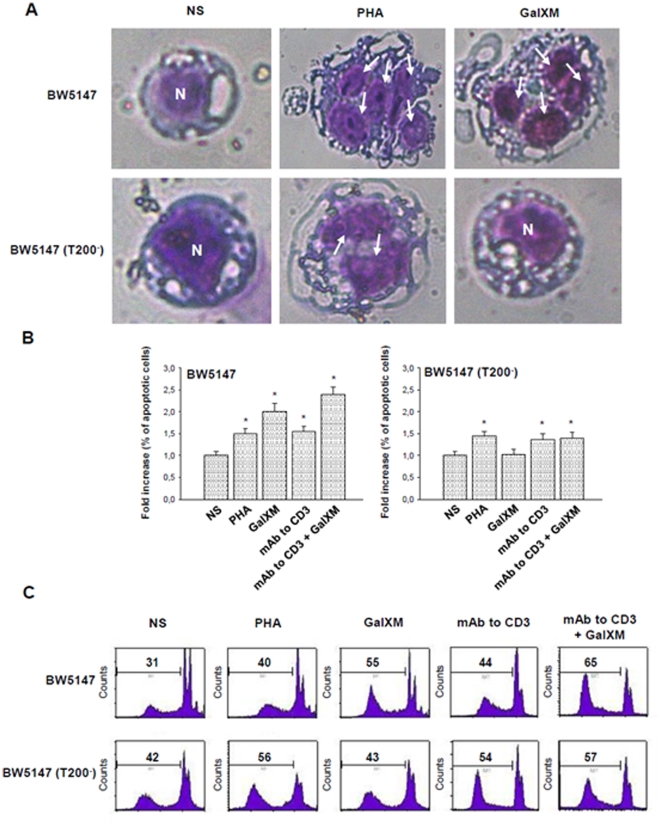
GalXM-induced apoptosis of BW5147 cells. (**A**) BW5147 and BW5147 (T200^−^) cells (both 1×10^6^/ml) were incubated for 18 h in the presence or absence (NS) of PHA (10 µg/ml) or GalXM (10 µg/ml). After incubation, cells were collected by cytospin and stained by Hemacolor. GalXM-treated cells exhibited altered morphology, surface blubs and nuclear fragmentation (arrows). N = cell nucleus; original magnification 40x. In selected experiments, cells were incubated for 18 h in the presence or absence (NS) of PHA (10 µg/ml), mAb to CD3 (1 µg/ml) or GalXM (10 µg/ml). After incubation, the percentage of cells undergoing apoptosis was evaluated by PI staining and analyzed using FACScan flow cytometry. Data are expressed as fold increase of percentage of apoptotic cells (**B**), or shown as FACScan histograms from one representative experiment out of seven with similar results (**C**). *, *p*<0.05 (treated *vs* untreated, n = 7). Error bars denote s.e.m.

In a previous study, we showed that GalXM activates the extrinsic and intrinsic apoptotic pathways through the interaction with T cell glycoreceptors. In particular, CD45 was primarily involved in Jurkat cells apoptosis while CD7 and CD43 mediated human T cell apoptosis [17]. In the present study, we investigated the effects of GalXM on CD45 requirement in T cell response. To this end, we used BW5147 (CD45^+/+^) and BW5147 (T200^−^) (CD45^−/−^) cell lines derived from BW5147 murine T cell lymphoma [18] routinely used for the study of the role of CD45 receptor in T cell response. Firstly, to verify the association of GalXM to both cell lines, BW5147 and BW5147 (T200^−^) cells were treated with GalXM-FLUOS. Results showed that GalXM association was observed in both cell lines after 30 min and 2 h of incubation (Fig. 1A). This result was confirmed by fluorescence microscopic analysis. Indeed, both cell types showed similar rate of GalXM association as observed in Figure 1B. The treatment of BW5147 and BW5147 (T200^−^) cells with native unlabelled GalXM significantly inhibited GalXM-FLUOS association (not shown). To analyze whether in these cells GalXM binds to CD45, we determined CD45 expression after GalXM addition. The results showed that treatment with GalXM for 30 min masked CD45 expression by 15% on BW5147 and this effect was still evident after 2 h of incubation, but was lost after 18 h (Fig. 2A). This effect was also shown with fluorescence histograms (Fig. 2B). BW5147 (T200^−^) cells were used as a negative control (Fig. 2B). In the analysis of CD45^+^/GalXM^+^ BW5147 cells we detected a significant increase in the percentage of double positive BW5147 but not BW5147 (T200^−^) cells after 30 min of GalXM-FLUOS treatment (Fig. 2C). The association of GalXM to CD45 was also demonstrated by data reported in Figure 2D. In BW5147 cells, GalXM colocalized with CD45 after 30 min of treatment. The overlap of the green light from GalXM-FLUOS and the red light from RPE-conjugated mAb to CD45 resulted in a yellow hue (Fig. 2D). Moreover, colocalization of GalXM with CD45 resulted in a subsequent segregation of CD45 to membrane patches after 2 h of incubation (Fig. 3A and B). BW5147 (T200^−^) was used as a negative control (Fig. 2D and Fig. 3). Previously, we demonstrated that GalXM was able to induce both the extrinsic and intrinsic apoptotic pathways in T cells through the interaction with the glycoreceptors, in particular through the interaction with CD45. To analyze the precise role of CD45 in GalXM-mediated apoptosis, BW5147 and BW5147 (T200^−^) cells were treated for 18 h with GalXM and apoptosis was evaluated. GalXM-treated BW5147 cells exhibited apoptotic cellular changes consisting of an altered morphology, characterized by surface blubs and nuclear fragmentation (Fig. 4A). Conversely, BW5147 (T200^−^) cells did not show any cellular changes in response to GalXM treatment (Fig. 4A). Moreover, a significant increase in the percentage of BW5147 apoptotic cells was evident 18 h after GalXM treatment, while this effect was absent in BW5147 (T200^−^) cells. This effect was also evident in cells activated with mAb to CD3 (Fig. 4B and 4C). PHA and mAb to CD3 were used as pro-apoptotic stimuli.

**Figure 5:**
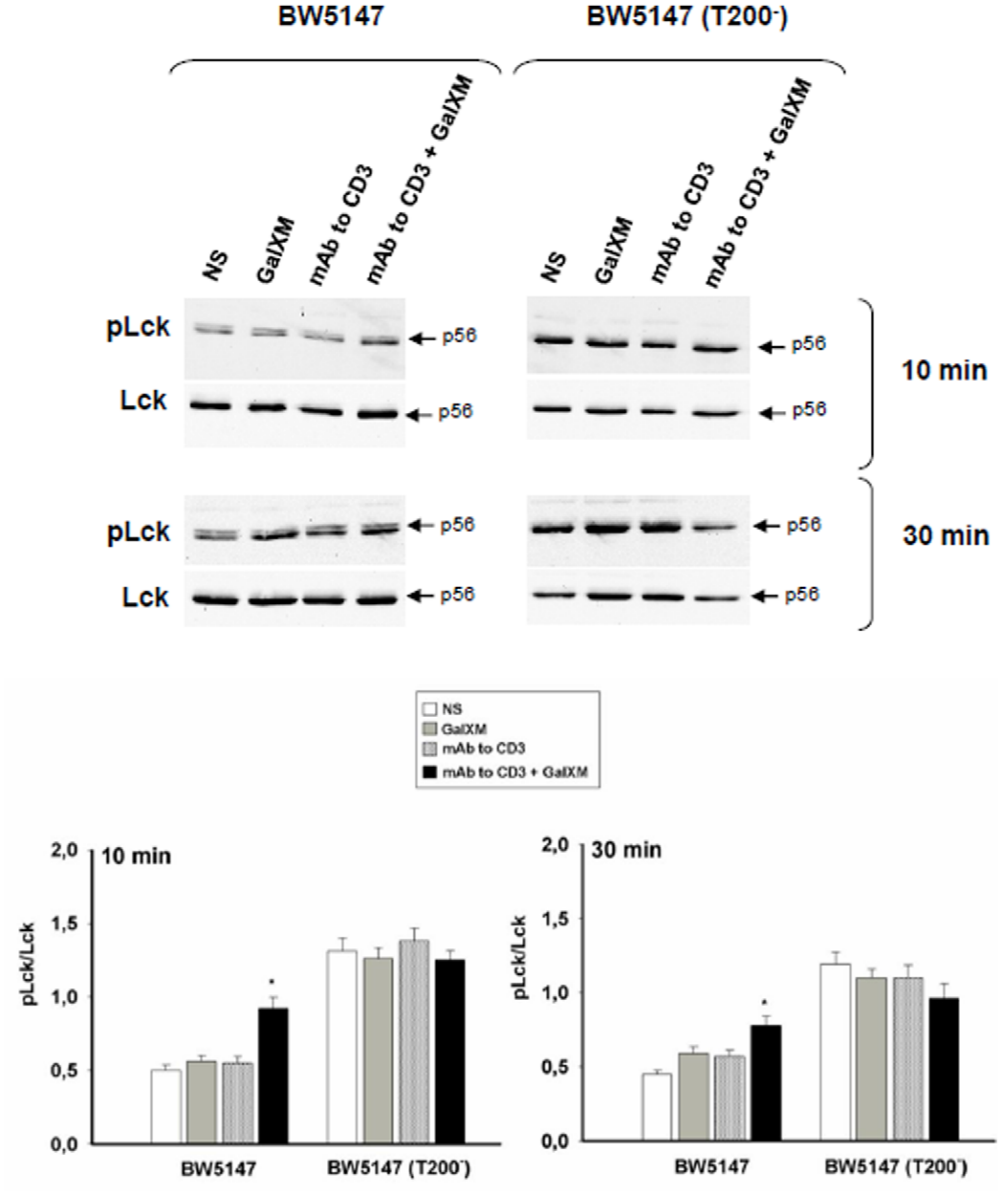
Phospho-Lck activation on BW5147 cells treated with GalXM. BW5147 and BW5147 (T200^−^) cells (both 5×10^6^/ml) were pre-activated for 30 min in the presence or absence (NS) of mAb to CD3 (1 µg/ml) and then incubated for 10 or 30 min in the presence or absence (NS) of GalXM (10 µg/ml). After incubation, cell lysates were analyzed by Western blotting. Membranes were incubated with antibodies to phospho-Lck and Lck. pLck was normalized against Lck. *, *p*<0.05 (mAb to CD3 plus GalXM treated *vs* untreated, n = 5). Error bars denote s.e.m.

Ligation of the T cell receptor (TCR) with the peptide antigen associated to major histocompatibility complex (MHC) molecules initiates a signal transduction cascade that ultimately leads to T cell activation [29]. Indeed, CD45 is a positive regulator of the TCR signaling and it is essential for signal transduction after an antigenic stimulation [29], [30]. The first event is activation of the Src family protein tyrosine kinases (SFKs) Fyn and Lck through the dephosphorylation of their crucial tyrosine residues mediated by CD45 [31], [32]. To understand whether GalXM influences TCR activation through its interaction with CD45, BW5147 cells were activated for 30 min with mAb to CD3 and then incubated for 10 or 30 min with GalXM. Our results showed that BW5147 cells stimulated with mAb to CD3 and then treated with GalXM exhibited an augmented expression of phosphorylated inactive form of Lck relative to cells only stimulated with mAb to CD3 (Fig. 5); conversely, BW5147 (T200^−^) cells showed a constitutive hyperphosphorylation of Lck [31] that was unaffected by the GalXM treatment (Fig. 5).

**Figure 6:**
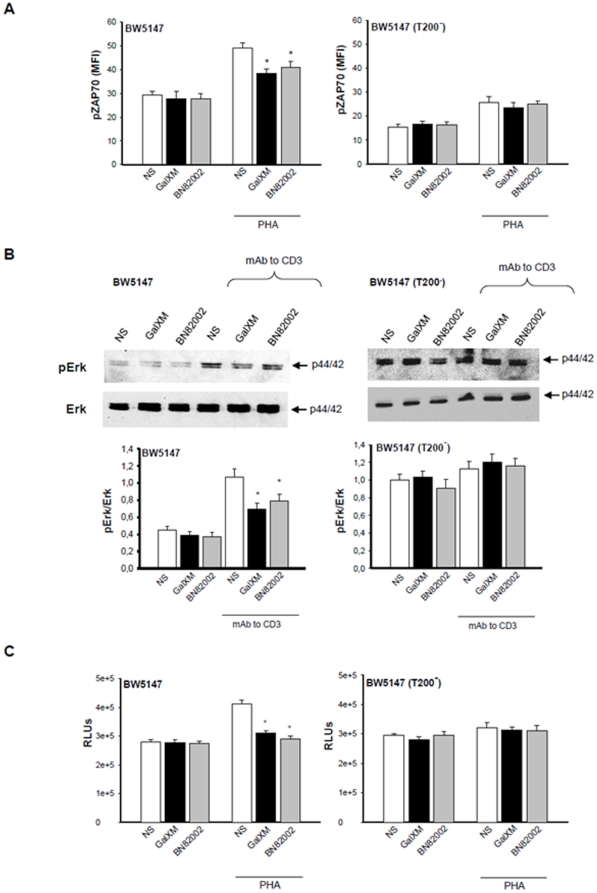
Phospho-ZAP70, phospho-Erk activation and proliferation of BW5147 cells treated with GalXM or BN82002. (**A**) BW5147 and BW5147 (T200^−^) cells (both 1×10^6^/ml) were pre-activated for 30 min in the presence or absence (NS) of PHA (10 µg/ml) and then incubated for 30 min in the presence or absence (NS) of GalXM (10 µg/ml) or BN82002 (6 µM). After incubation cells were labelled with antibody to phospho-ZAP70 and then analyzed by FACScan flow cytometry. The mean of fluorescence intensity (MFI) of labelled cells is shown as a histogram. *, *p*<0.05 (treated *vs* untreated, n = 7). (**B**) BW5147 and BW5147 (T200^−^) cells (both 5×10^6^/ml) were pre-activated for 30 min in the presence or absence (NS) of mAb to CD3 (1 µg/ml) and then incubated for 30 min in the presence or absence (NS) of GalXM (10 µg/ml) or BN82002 (6 µM). After incubation, cell lysates were analyzed by Western blotting; membranes were incubated with antibodies to phospho-Erk1/2 and Erk1/2. pErk was normalized against Erk. *, *p*<0.05 (treated *vs* untreated, n = 7). Error bars denote s.e.m. (**C**) BW5147 and BW5147 (T200^−^) cells (both 1×10^6^/ml) were pre-activated for 30 min in the presence or absence (NS) of PHA (10 µg/ml) and then incubated for 48 h in the presence or absence (NS) of GalXM (10 µg/ml) or BN82002 (6 µM). After incubation, cell proliferation was evaluated by ViaLight Plus Kit. *, *p*<0.05 (treated *vs* untreated, n = 7). Error bars denote s.e.m.

Lck activation leads to ZAP70 phosphorylation [31]. ZAP70 is a member of the Syk family kinase predominantly involved in TCR signaling initiation and subsequent T cell activation [33], therefore we analyzed the effect of GalXM treatment on ZAP70 activation. The results showed a decrease in the mean fluorescence intensity (MFI) of phospho-ZAP70 in BW5147 cells pre-activated with PHA and treated with GalXM, compared to cells treated only with PHA (Fig. 6A). The GalXM treatment did not reduce the up-regulation of phospho-ZAP70 in activated BW5147(T200^−^) cells (Fig. 6A).

Given that signaling molecules such as Lck and the mitogen-activated protein kinases (MAPKs) cooperate to produce TCR activation signaling [29], a possible involvement of Erk1/2 activation in our experimental system was investigated. A decrease of phosphorylated active form of Erk1/2 was observed in pre-activated BW5147 cells treated with GalXM, compared to GalXM untreated cells (Fig. 6B). No modulation of Erk1/2 phosphorylation was observed in BW5147 (T200^−^) cells treated or untreated with GalXM (Fig. 6B).

A CD45-phosphatase activity inhibitor, BN82002, was used as a control to mimic the inhibitory effect of GalXM on CD45 activity. The results showed that in pre-activated BW5147 cells, BN82002 reduced phospho-ZAP70 and phospho-Erk1/2 activation. Phospho-ZAP70 expression and phospho-Erk1/2 activation were not modulated by treatment with GalXM or BN82002 in BW5147 (T200^−^) cells (Fig. 6A and 6B).

**Figure 7:**
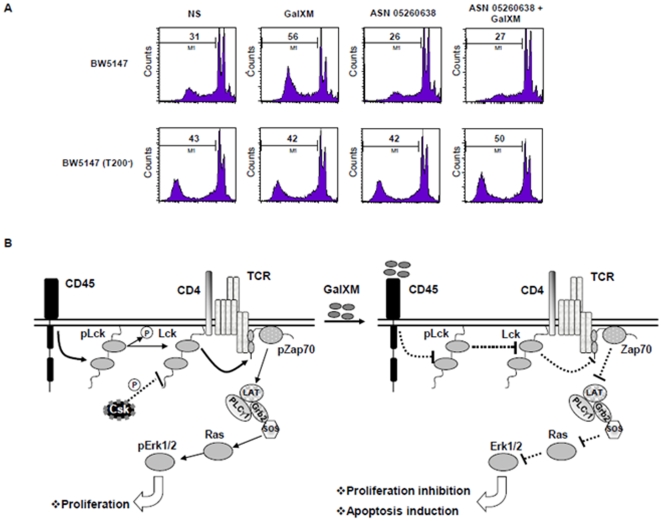
Blocking of CD45 phosphotase activity induces apoptosis and inhibits proliferation of BW5147 cells treated with GalXM. (**A**) BW5147 and BW5147 (T200^−^) cells (both 1×10^6^/ml) were pre-treated for 10 min with ASN 05260638 (20 µM) and then incubated for 18 h in the presence or absence (NS) of GalXM (10 µg/ml). After incubation, the percentage of cells undergoing apoptosis was evaluated by PI staining and analyzed using FACScan flow cytometry. Data are expressed as FACScan histograms from one representative experiment out of five with similar results. (**B**) Schematic representation of GalXM effect on BW5147 cells. GalXM induces immunosuppressive effects on T cells through Lck inactivation. This effects occurs after GalXM association to CD45, which inhibits its dephosphatase activity.

Finally we analyzed the biological effects of GalXM on BW5147 cell proliferation using BN82002 as a control. The augmented proliferation in cells pre-activated with PHA was inhibited by the treatment with GalXM or BN82002 (Fig. 6C). Again, this effect was not observed in BW5147 (T200^−^) cells (Fig. 6C). Furthermore, we sought to determine if GalXM association to CD45 induced apoptosis of BW5147 cells through the inhibition of its phosphatase activity. Thus, we treated cells with the Csk inhibitor ASN 05260638, to induce a state of dephosphorylation of Lck, and then the cells were incubated for 18 h in the presence or absence of GalXM. Pre-treatment of BW5147 cells with ASN 05260638 inhibited the induction of apoptosis mediated by GalXM (Fig. 7A). BW5147 (T200^−^) cells were used as a control (Fig. 7A). The inhibition of CD45 phosphatase activity, induced by the association of GalXM to CD45, resulted in widespread T cell damage through the downregulation of Lck activity. A model of the proposed mechanisms of inhibitory effects induced by GalXM through CD45 is proposed (Fig. 7B).

## Discussion

We previously reported that GalXM induces apoptosis of T cells by the induction of Fas and FasL up-regulation that leads to the activation of extrinsic and intrinsic apoptotic pathways through the cleavage and recruitment of caspase-8, Bid, caspase-9 and effector caspases 3, 6 and 7. These effects are dependent on glycoreceptor engagement by GalXM [17]. Furthermore, glycoreceptors have also been reported to be involved in the inhibition of T cell proliferation [34], [35]. In this study we demonstrate that: i) GalXM interaction with T cells was independent of CD45 expression; ii) CD45 was identified as the primary molecular target for GalXM-induced T cell modulatory effects; iii) immunoregulation required the interaction of CD45 with protein tyrosine kinases such as Lck and ZAP70; iv) GalXM/CD45 interaction inhibited Erk1/2 phosphorylation, this effect being directly involved in the inhibition of T cell activation; and v) GalXM/CD45 interaction induced apoptosis through the inhibition of CD45 phosphatase activity.

Furthermore, in this study we provide evidence of direct association of GalXM with T cells. Even though the GalXM regulation of biological function of T cells was clearly apparent, a relatively low percentage of immune cells were found to be associated with GalXM. A possible explanation includes the low dose of GalXM used (10 µg/ml), steric hindrance of GalXM, or both of these reasons. The GalXM-induced effects include inhibition of T cell activation and induction of T cell death. We previosly demonstrated that the apoptosis induced by GalXM is mediated by upregulation of Fas/FasL expression on T cells [17]. In this study we identify the CD45 activation pathway involved in apoptosis induction and in direct inhibition of T cell activation.

LPS could possibly contaminate GalXM, but there are at least two reasons to exclude this: the first is the negative results obtained using the *Limulus* test, the second is the inhibitory nature of GalXM, which is in contradistinction to the effects of LPS. The absence of measurable LPS combined with the GalXM-mediated immunosuppressive effects essentially rules out the possibility that LPS contributed to the observed results. The induction of T cell apoptosis mediated by GalXM might be, in several aspects, similar to that induced by galectins. Galectins are a family of carbohydrate-associated mammalian proteins with an affinity for β-galactosides that positively or negatively regulates apoptosis [35], [36], [37]. Galectin-1 and galectin-3, the most ubiquitously expressed members of the galectins family, are expressed in several tissues including thymus and lymph nodes [36]. These two lectins recognize discrete sets of oligosaccharide ligands. The similarities between galectins and GalXM include a) regulation of Fas-mediated death pathways and impairment of T cell activation; b) association to T cells and induction of their death [38], [39]; and c) CD45 engagement and related biological effects.

Multiple isoforms of CD45 exist as a result of the alternative RNA splicing [3], [40], this mechanism implies concurrent changes in glycosylation, for example activated T cells express CD45 that are hyposialylated compared to resting T cells [41].

The fact that GalXM is able to induce apoptotic and suppressive effects particularly in pre-activated T cells suggests that GalXM could preferentially bind to hyposialylated, low molecular weight CD45 isoforms (CD45RO). In turn this suggests that CD45 glycosylation regulates the susceptibility to GalXM-mediated T cell death. Furthermore, given that CD45 is also expressed in B cells, the mechanisms of depletion of B cells and of immunological paralysis recently described by De Jesus *et al.* [15] could be related to the alteration of signaling cascade of BCR-induced activation, caused by CD45 engagement by GalXM [42].

CD45 interaction with its ligand can induce dimerization [43]. The negative regulation of CD45, associated with inhibition of its phosphatase activity, has been ascribed to CD45 dimerization. Indeed, CD45RO homodimerizes more easily than high molecular weight isoforms, leading to inhibition of phosphatase activity [40]; this model reinforces the concept that GalXM interacts particularly with CD45RO.

Cross-linking of CD45 has been implicated in apoptosis induction [44], [45] and a role for CD45RO has been suggested [46]. Given that the major substrate of CD45 is Lck, one might posit that it is implicated in the apoptotic pathway activation. There are studies reporting that CD45 and Lck are not involved in Fas-induced apoptosis, because Fas-induced apoptosis was similar in wild type and cells deficient in CD45 or Lck [47], [48]. However, it is noteworthy that the cells used in some of those studies were not completely deficient in CD45 [49], and therefore, these studies were not conclusive. In a recent paper we demonstrated that GalXM induces Fas/FasL expression on T cells and this phenomenon is greatly responsible of apoptosis induction. Despite convincing evidence for CD45 implication in apoptosis regulation, very little is known about the mechanisms and pathways involved in this process. A relationship between CD45 expression and apoptosis activation was discussed in this paper. We demonstrated that GalXM induced apoptosis of T cells through inhibition of CD45 phosphatase activity. However, we were able to observe an increase of GalXM-induced phospho-Lck only in activated cells. Indeed, phospho-Lck was tested after 10 and 30 min, while apoptosis induction was evidenced after 18 h. Thus the kinetics and quantity of phospho-Lck activation could be different in inactivated and activated cells and, as a consequence, observable, at least at 10 and 30 min, only in activated cells. GalXM/CD45 interaction clusters and segregates CD45 and it is possible that this segregation is regulated by attachment to the cytoskeleton through the linker protein fodrin [41]. An association also exists between DNA fragmentation and Lck phosphorylation. Indeed, CD45 initiates T cell antigen signal transduction by dephosphorylating Lck [50], [51]. The hypothesis that Lck is implicated in induction of apoptosis is supported by the constitutive hyperphosphorylation of Lck observed in CD45^−/−^ cells that are refractory to GalXM-induced apoptosis.

This in turn is supported by recent studies demonstrating that the tyrosine kinase Lck is involved in the regulation of apoptosis, particularly in the mitochondrial-induced pathway [52], [53]. Given that GalXM activation of this pathway was previously observed [17], the results reported in this paper support this hypothesis and suggest the possible implication of ZAP70 substrate.

The correlation between Lck phosphorylation and apoptosis induction is supported by the fact that the induction of dephosphorylation of Lck, results in inhibition of apoptosis GalXM-induced. Moreover, a CD45-phosphatase activity inhibitor, that should mirror the GalXM signal pathway, inhibits T cell activation. Building on these results one could posit that GalXM could damage T cells by inducing apoptosis as well as by inhibiting other biological functions. More investigations need to be done to clarify the relationship between these effects.

MAPK involvement in apoptosis has been demonstrated [29], [44]. Erk1/2 activation can protect cells from cisplatinum-induced apoptosis [54], and Erk1/2 is also important in recruiting cFos to the nucleus, where the activation of transcription factor AP-1 regulates cell cycle and apoptosis [55]. In addition, Erk1/2 activation can occur via Lck [29].

The evidence for the involvement of MAPK in apoptosis is contradictory and depends on the cell type and the type of apoptosis inducer [29], [44]. Here we report that Erk1/2 is down-regulated by GalXM stimulation exclusively in CD45 wild type cells, while hyperphosphorylation of Erk1/2 is observed in CD45^−/−^ cells. Thus, we hypothesize that Erk1/2 is implicated in the induction of GalXM-mediated apoptosis.

Indeed, GalXM induces apoptosis as well as inhibition of T cell activation. However, we cannot distinguish the involvement of the signaling mechanism in these two processes. It has been reported that CD45 is sequentially cleaved by serine/metalloproteinase and γ-secretase during its activation by fungal stimuli. CD45 cleavages release a fragment of the CD45 cytoplasmic tail (ct-CD45). Soluble ct-CD45 selectively binds to pre-activated T cells and inhibits T cell proliferation [56]. Given that GalXM is a fungal antigen [11], [12] it might be that soluble ct-CD45 is released, in the presence of GalXM, and this might contribute to the inhibition of T cell activation.

With regards to cryptococcal pathogenesis our results detail the powerful immunosuppressive action of GalXM through its effects on CD45-related apoptosis. This mechanism may be responsible for the widespread disruption of the immune function that follows GalXM administration [13], [14], [15], [16], and for the immunosuppression that accompanies cryptococcosis.
